# *trans*-(2,2′-Bipyrimidine)diiodido(isopropoxido)oxidorhenium(V)

**DOI:** 10.1107/S1600536809052556

**Published:** 2009-12-12

**Authors:** Andrzej Kochel

**Affiliations:** a Faculty of Chemistry, University of Wrocław, 14 Joliot-Curie St, 50-383 Wrocław, Poland

## Abstract

In the title compound, [Re(C_3_H_7_O)I_2_O(C_8_H_6_N_4_)], the Re^V^ atom adopts a distorted octa­hedral ReI_2_O_2_N_2_ geometry, with the O atoms in a *trans* conformation and the I atoms in a *cis* conformation. Two intra­molecular C—H⋯I contacts occur. The crystal structure is stabilized by inter­molecular C—H⋯O, C—H⋯N and C—H⋯I hydrogen bonds.

## Related literature

For related structures and for further discussion of rhenium structural chemistry, see: Abrahams *et al.* (2005 ▶, 2007 ▶); Abram *et al.* (1995 ▶); Ciani *et al.* (1983 ▶); Gerber *et al.* (2004 ▶). Graziani *et al.* (1985 ▶); Herrman *et al.* (1990 ▶); Irmler *et al.* (1991 ▶); Lebuis *et al.* (1993 ▶); Mrozinski *et al.* (2002 ▶); Quintal *et al.* (2000 ▶); Schmidt-Brucken & Abram (2000 ▶). For further synthetic details, see: Watt & Thompson (1963 ▶).
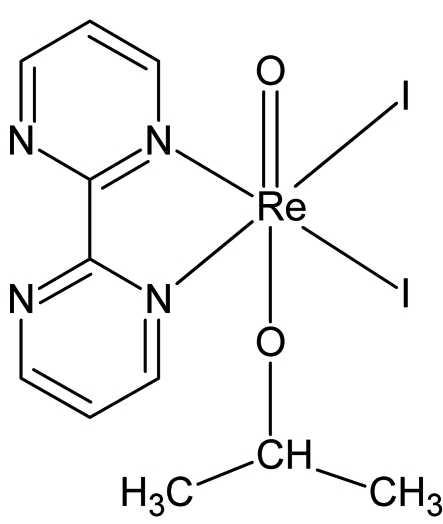


## Experimental

### 

#### Crystal data

[Re(C_3_H_7_O)I_2_O(C_8_H_6_N_4_)]
*M* *_r_* = 673.26
Monoclinic, 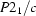

*a* = 10.3973 (3) Å
*b* = 10.9046 (3) Å
*c* = 15.6341 (6) Å
β = 117.616 (2)°
*V* = 1570.63 (9) Å^3^
*Z* = 4
Mo *K*α radiation
μ = 11.67 mm^−1^
*T* = 100 K
0.12 × 0.11 × 0.03 mm

#### Data collection

Oxford Diffraction KM-4-CCD diffractometer
Absorption correction: analytical (*CrysAlis RED*; Oxford Diffraction, 2007 ▶) *T*_min_ = 0.456, *T*_max_ = 0.611
15982 measured reflections
2776 independent reflections
2288 reflections with *I* > 2σ(*I*)
*R*_int_ = 0.050

#### Refinement

*R*[*F* ^2^ > 2σ(*F* ^2^)] = 0.029
*wR*(*F* ^2^) = 0.056
*S* = 1.03
2776 reflections
183 parameters
H-atom parameters constrained
Δρ_max_ = 1.85 e Å^−3^
Δρ_min_ = −1.34 e Å^−3^

### 

Data collection: *CrysAlis CCD* (Oxford Diffraction, 2007 ▶); cell refinement: *CrysAlis RED* (Oxford Diffraction, 2007 ▶); data reduction: *CrysAlis RED*; program(s) used to solve structure: *SHELXS97* (Sheldrick, 2008 ▶); program(s) used to refine structure: *SHELXL97* (Sheldrick, 2008 ▶); molecular graphics: *DIAMOND* (Brandenburg & Putz, 2005 ▶); software used to prepare material for publication: *SHELXL97*.

## Supplementary Material

Crystal structure: contains datablocks global, I. DOI: 10.1107/S1600536809052556/hb5231sup1.cif

Structure factors: contains datablocks I. DOI: 10.1107/S1600536809052556/hb5231Isup2.hkl

Additional supplementary materials:  crystallographic information; 3D view; checkCIF report

## Figures and Tables

**Table:** 

Re1—O1	1.698 (4)
Re1—O2	1.861 (5)
Re1—N1	2.175 (5)
Re1—N2	2.183 (6)
Re1—I1	2.7176 (5)
Re1—I2	2.7235 (5)

**Table:** 

N1—Re1—N2	76.5 (2)

**Table 2:** Hydrogen-bond geometry (Å, °)

*D*—H⋯*A*	*D*—H	H⋯*A*	*D*⋯*A*	*D*—H⋯*A*
C1—H1⋯I1	0.95	3.04	3.697 (7)	127
C6—H6⋯I2	0.95	3.05	3.709 (7)	128
C5—H5⋯N4^i^	0.95	2.58	3.504 (9)	163
C6—H6⋯O1^ii^	0.95	2.53	3.193 (9)	127
C9—H9⋯I1^iii^	1.00	3.02	3.834 (8)	139

Symmetry codes: (i) 
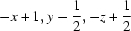
; (ii) 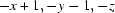; (iii) 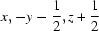.

**Fig. 1.**
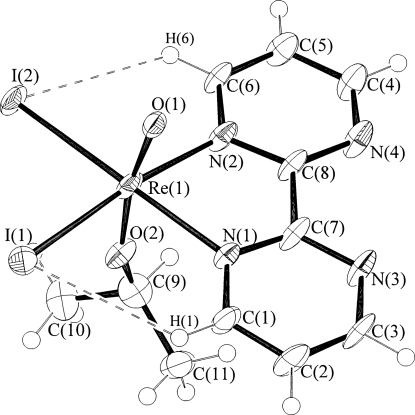
View of (I) showing 50% displacement ellipsoids (H atoms as spheres of arbitrary radius).

**Fig. 2.**
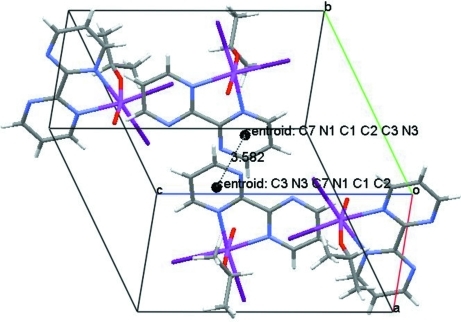
The crystal packing in (I) viewed down [100].

**Fig. 3.**
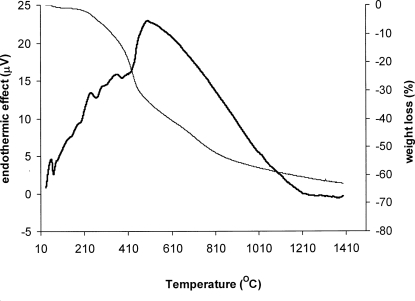
Corresponding TG/DTA curves for 1. The thicker line denotes the thermal effect, the thinner line denotes the weight loss curve.

## supplementary crystallographic information

### Comment

Re^V^ complexes have been research object for many authors during the last years. Complexes of general formula [ReO*X*_2_(C_5_H_4_N)CH(O)CH_2_(C_5_H_4_N)] (*X* = Cl, I) were obtained by a reaction of *trans*-[ReOCl_3_(PPh_3_)_2_] and *trans*-[ReOI_2_(OEt)(PPh_3_)_2_] with *cis*-1,2-di-(2-pyridyl)ethylene (DPE) in ethanol and in benzene on air. The coordinated DPE ligand undergoes addition of water at the ethylene carbon atoms, and the (C_5_H_4_N)CH(O)CH_2_(C_5_H_4_N) moiety acts as terdentate N,*O*,*N*-donor ligand. X-ray crystal structures of both complexes have been determined and show distorted octahedral geometry around the rhenium(V) centre (Abrahams *et al.*, 2005). Complexes *cis*-[ReO*X*_2_(msa)(PPh_3_)] [*X* = Cl(1), I(2)] were prepared from *trans*-[ReOCl_3_(PPh_3_)_2_] or *trans*-[ReOI_2_(OEt)(PPh_3_)_2_] in reaction with 2-(1-iminoethyl)phenol (Hmsa) in acetonitrile. X-ray crystal structure shows, that the bonding distances and angles in 1 and 2 are nearly identical, and that the two halide ligands in each complex are coordinated *cis* to each other in the equatorial plane *cis* to the oxido group. Rhenium(V) complexes with two iodido ligands *cis* to each other are rare (Abrahams *et al.*, 2007). Rhenium(V) complexes with *trans*-O—Re-(2-propoxido) core are known. Rhenium(V) complex with 2-propoxido ligand of formula [ReOCl_2_(C_3_H_7_O)(Ph_3_P)_2_] has been synthesized (Abram *et al.* 1995), and the other one, [ReClO(C_3_H_7_O)PPh_3_L] (where *L* = pyridine-2-thiolato ligand), was obtained (Schmidt-Brucken & Abram, 2000). This paper contains a report on the synthesis and structure of the first characterized [ReOI_2_(C_3_H_7_O)(2–2?bipyrimidine)] 1 complex with ReI_2_ core and 2-propoxido ligand. Fig. 1 presents the view of molecular structure of compound 1, the compound crystallizes in monoclinic crystal system in *P*2_1_/*c* space group. The compound was obtained in the synthesis with (NH_4_)_2_ReI_6_ and 2,2'-bipyrimidine as substrates in a mixture of 2-propanol and acetone. The environment around the metal center is a distorted octahedron, with two iodido ligands, 2-propoxido ligand and 2,2'-bipyrimidine ligand coordinated *via* nitrogen atoms. The Re—I bond lengths are Re1—I1 2.7176 (5) Å, Re1—I2 2.7235 (5) Å, respectively,similar Re—I distances were observed in 2-(2-aminophenyl)ethanolato-*N*,*O*)-bis(iododo)-oxido- triphenylphosphine-rhenium(V) and diodido-(3-hydroxidopicolinato)-oxido-)-triphenylphosphine-rhenium(II) (Gerber *et al.*, 2004, Quintal *et al.*, 2000). On the other hand, the Re1—O1 1.698 (4) Å bond length is characteristic for monooxido-rhenium(V) complexes (on average 1.69%A), which is in agreement with the situation in the comparable complexes (Graziani *et al.*, 1985, Lebuis *et al.*, 1993). The O atom from 2-propoxido ligand is coordinated to the rhenium atom with Re—O2 1.861 (5) Å; this bond is remarkably short with the same multiple bond character as discussed (Ciani *et al.* 1983). The O—Re—O unit is nearly linear with an angle of 169.69 (3)°. Moreover, a molecule of 2,2'-bipyrimidine is coordinated with Re—N bond length of Re1—N1 2.175 (5) %A, Re1—N2 2.183 (6) Å, which is comparable to the previously investigated *trans*-[ReCl_4_(py)_2_] (Mrozinski *et al.*, 2002, Herrman *et al.*, 1990). Re atom lies within the plane of I1, I2, N1, N2 atoms, the N1—Re1—O1 and N2—Re1—O1 angles are 88.63 (19), 86.93 (14) and N1—Re1—O2, N2—Re1—O2 83.37 (14) and 84.87 (15) and are directed in side to 2,2'-bipyrimidine. The rings of the 4,4'-bpy ligand are coplanar with the plane defined by the I ligands, one of the Re—N bond is shorter (Re1—N1 2.175 (5) Å, Re1—N2 2.183 (6) Å), which may be a result of iodido ligand presence. The molecule conformation is stabilized by intramolecular hydrogen bonds C(1)—H(1)···I(1) and C(6)—-H(6)···I(2), as well as by the intermolecular hydrogen bonds of C—H···O and C—H···N type. All hydrogen bonds are summarized in Table 2. In the crystal structure packing along [100] and [010] a layered arrangement of the molecules could be observed. The crystal structure packing viewed along [100] direction is illustrated in Fig. 2. In the structure the stacking interactions are observed. pi-pi stacking interactions between the 2,2'-bypirymidine rings contribute to forming a supramolecular network structure (Fig. 2). The centroid-centroid distance of the adjacent aromatic rings is about 3.58%A, indicating a normal pi-pi interaction. The corresponding TG-DTA curves (the measurement was carried out under nitrogen atmosphere) for the title compound (Fig. 3) show a three-step decomposition process and that the compound is very stable during heating. In the first step of the thermal decomposition process weight loss in the temperature range 242–247°C of 1.39% (calc. 1.43%) is observed. In DTA curve, this decomposition is visible as an endothermic peak at temperature 247°C, indicating the presence of coordinated 4.4,-bipyrimidine (corresponding to the weight loss of 1.39% in thermal decomposition, which is in agreement with the calculated value of 1.43%). Further weight losses correspond to decomposition of further compound parts, they are continuous and are difficult for unambiguous interpretation (decomposition of all compound constituents except for rhenium part). Another peak at 800°C is also observed, however, it is difficult to interpret. It is possible, that the decomposition process leads to rhenium oxides. Similar decomposition process through many intermediate stages has been observed for (NH_4_)_2_[Re_3_Cl_12_] (Irmler *et al.*, 1991). In conclusion, an interesting rhenium(V) *trans*-2,2,-bipyrimidine-diiodido-oxido-(2-propoxido)-rhenium(V) complex with 2-propoxido ligand was obtained. The crystal structure is stabilized by hydrogen bonding interactions. This is the first structural report confirming the existence of a iodido rhenium complex with an aromatic amine and 2-propoxido ligand in coordination sphere.

### Experimental

A mixture of (NH_4_)_2_[ReI_6_] (prepared according to the literature procedure (Watt & Thompson, 1963) (0.12 g) and 0.30 g of 2,2,-(bipyrimidine) (from Aldrich) was added to a mixture of 2-propanol/acetone (1:1) (50 *cm*3) and then was stirred at 40° for about 4 h, the color of the solution was dark green. The solution was left for slow crystallization by evaporation under parafilm. The mixture was kept at room temperature for crystallization. After five days the pale green plate-shaped crystals were obtained. Anal. Calc. For: [ReOI_2_(C_3_H_7_O)(2,2,bipyrimidine)] C 19.62, H 2.08, N 8.32, I 37.69%: found C 19.01, H 1.89, N 8.15, I 37.50%. Selected IR data (KBr): 2925 (*m*), 2854 (*m*), 1717 (*m*), 1631 (*m*), 1610 (w), 1558 (*s*), 1464 (*m*), 1406 (*s*),1378 (*m*), 1271 (w), 1193 (w), 1191 (w), 978 (w), 908(w), 807 (w),645 (*m*), 561 (w), 535 (w), 452 (*s*), 390 (w), 358(w), 315 (*s*), 162 (*versus*), 131 (*s*), 74 (w).

TG–DTA Thermogravimetric measurements were carried out using a TG–DTA SETSYS 16Y18 device under nitrogen atmosphere for a sample placed in Al_2_O_3_ crucible. The investigated temperature range was from room temperature to 1400°C at 10°C_/_min. temperature changes rate. IR spectra The room temperature FT—IR spectra of polycrystalline samples were measured by means of the Bruker IFS-66 instrument.

### Refinement

The H atoms were generated geometrically and refined using a riding model, with C—H = 0.95 Å and *U*_iso_(H) = 1.2 *U*_eq_(C). The highest peak of 0.82 electrons at the difference Fourier map was situated near the Re atom.

### Crystal data

**Table:**

[Re(C_3_H_7_O)I_2_O(C_8_H_6_N_4_)]	*F*(000) = 1216
*M**_r_* = 673.26	*D*_x_ = 2.847 Mg m^−^^3^
Monoclinic, *P*2_1_/*c*	Mo *K*α radiation, λ = 0.71073 Å
Hall symbol: -P 2ybc	Cell parameters from 2115 reflections
*a* = 10.3973 (3) Å	θ = 2.9–25.1°
*b* = 10.9046 (3) Å	µ = 11.67 mm^−^^1^
*c* = 15.6341 (6) Å	*T* = 100 K
β = 117.616 (2)°	Plate, pale green
*V* = 1570.63 (9) Å^3^	0.12 × 0.11 × 0.03 mm
*Z* = 4	

### Data collection

**Table:**

Oxford Diffraction KM-4-CCD diffractometer	2776 independent reflections
Radiation source: fine-focus sealed tube	2288 reflections with *I* > 2σ(*I*)
graphite	*R*_int_ = 0.050
/w scans	θ_max_ = 25.1°, θ_min_ = 2.9°
Absorption correction: analytical (*CrysAlis RED*; Oxford Diffraction, 2007)	*h* = −12→12
*T*_min_ = 0.456, *T*_max_ = 0.611	*k* = −11→12
15982 measured reflections	*l* = −18→18

### Refinement

**Table:**

Refinement on *F*^2^	Primary atom site location: structure-invariant direct methods
Least-squares matrix: full	Secondary atom site location: difference Fourier map
*R*[*F*^2^ > 2σ(*F*^2^)] = 0.029	Hydrogen site location: inferred from neighbouring sites
*wR*(*F*^2^) = 0.056	H-atom parameters constrained
*S* = 1.02	*w* = 1/[σ^2^(*F*_o_^2^) + (0.0251*P*)^2^ + 3.8665*P*] where *P* = (*F*_o_^2^ + 2*F*_c_^2^)/3
2776 reflections	(Δ/σ)_max_ = 0.001
183 parameters	Δρ_max_ = 1.85 e Å^−^^3^
0 restraints	Δρ_min_ = −1.34 e Å^−^^3^

### Special details

**Table:**

Geometry. All e.s.d.'s (except the e.s.d. in the dihedral angle between two l.s. planes) are estimated using the full covariance matrix. The cell e.s.d.'s are taken into account individually in the estimation of e.s.d.'s in distances, angles and torsion angles; correlations between e.s.d.'s in cell parameters are only used when they are defined by crystal symmetry. An approximate (isotropic) treatment of cell e.s.d.'s is used for estimating e.s.d.'s involving l.s. planes.
Refinement. Refinement of *F*^2^ against ALL reflections. The weighted *R*-factor *wR* and goodness of fit *S* are based on *F*^2^, conventional *R*-factors *R* are based on *F*, with *F* set to zero for negative *F*^2^. The threshold expression of *F*^2^ > σ(*F*^2^) is used only for calculating *R*-factors(gt) *etc*. and is not relevant to the choice of reflections for refinement. *R*-factors based on *F*^2^ are statistically about twice as large as those based on *F*, and *R*- factors based on ALL data will be even larger.

### Fractional atomic coordinates and isotropic or equivalent isotropic displacement parameters (Å^2^)

**Table:**

	*x*	*y*	*z*	*U*_iso_*/*U*_eq_	
Re1	0.26746 (3)	−0.33250 (2)	−0.10973 (2)	0.02512 (9)	
I1	0.13432 (6)	−0.31460 (4)	−0.30538 (3)	0.03659 (14)	
I2	0.22482 (5)	−0.57955 (4)	−0.12777 (3)	0.03021 (13)	
O1	0.4380 (5)	−0.3317 (4)	−0.0984 (3)	0.0228 (10)	
O2	0.0941 (5)	−0.3139 (4)	−0.1035 (3)	0.0317 (11)	
N1	0.2890 (6)	−0.1369 (5)	−0.0790 (4)	0.0228 (12)	
N2	0.3689 (7)	−0.3247 (5)	0.0476 (4)	0.0281 (13)	
N3	0.3837 (7)	0.0063 (5)	0.0504 (4)	0.0293 (14)	
N4	0.4828 (7)	−0.1874 (5)	0.1792 (4)	0.0316 (15)	
C1	0.2343 (8)	−0.0453 (6)	−0.1436 (5)	0.0324 (17)	
H1	0.1832	−0.0636	−0.2105	0.039*	
C2	0.2519 (9)	0.0735 (6)	−0.1134 (5)	0.0365 (19)	
H2	0.2114	0.1388	−0.1583	0.044*	
C3	0.3303 (8)	0.0959 (6)	−0.0157 (5)	0.0328 (18)	
H3	0.3473	0.1786	0.0057	0.039*	
C4	0.5150 (9)	−0.2833 (6)	0.2389 (5)	0.0342 (18)	
H4	0.5655	−0.2690	0.3065	0.041*	
C5	0.4775 (8)	−0.4035 (6)	0.2062 (5)	0.0320 (17)	
H5	0.5002	−0.4704	0.2498	0.038*	
C6	0.4065 (8)	−0.4201 (6)	0.1084 (5)	0.0273 (16)	
H6	0.3834	−0.5009	0.0830	0.033*	
C7	0.3584 (8)	−0.1071 (6)	0.0158 (5)	0.0291 (17)	
C8	0.4078 (8)	−0.2118 (6)	0.0862 (5)	0.0261 (16)	
C9	−0.0043 (9)	−0.2989 (8)	−0.0654 (6)	0.045 (2)	
H9	0.0494	−0.3159	0.0055	0.054*	
C10	−0.1237 (9)	−0.3889 (8)	−0.1089 (6)	0.043 (2)	
H10A	−0.1895	−0.3648	−0.1754	0.065*	
H10B	−0.1773	−0.3915	−0.0713	0.065*	
H10C	−0.0834	−0.4703	−0.1087	0.065*	
C11	−0.0531 (8)	−0.1652 (6)	−0.0783 (6)	0.0382 (18)	
H11A	0.0309	−0.1123	−0.0416	0.057*	
H11B	−0.1249	−0.1540	−0.0549	0.057*	
H11C	−0.0966	−0.1434	−0.1469	0.057*	

### Atomic displacement parameters (Å^2^)

**Table:**

	*U*^11^	*U*^22^	*U*^33^	*U*^12^	*U*^13^	*U*^23^
Re1	0.03734 (18)	0.00899 (14)	0.03104 (16)	0.00073 (12)	0.01756 (13)	0.00019 (11)
I1	0.0430 (3)	0.0213 (3)	0.0286 (2)	0.0025 (2)	0.0023 (2)	0.0026 (2)
I2	0.0453 (3)	0.0095 (2)	0.0337 (3)	−0.00078 (19)	0.0165 (2)	−0.00140 (18)
O1	0.031 (3)	0.015 (2)	0.022 (2)	0.0001 (19)	0.011 (2)	−0.0019 (19)
O2	0.041 (3)	0.016 (2)	0.041 (3)	0.002 (2)	0.022 (3)	−0.001 (2)
N1	0.030 (3)	0.013 (3)	0.028 (3)	0.001 (2)	0.016 (3)	0.000 (2)
N2	0.045 (4)	0.012 (3)	0.034 (3)	0.001 (3)	0.025 (3)	0.001 (3)
N3	0.056 (4)	0.012 (3)	0.036 (3)	−0.005 (3)	0.035 (3)	−0.004 (3)
N4	0.065 (4)	0.018 (3)	0.027 (3)	−0.003 (3)	0.034 (3)	−0.001 (2)
C1	0.049 (5)	0.016 (4)	0.029 (4)	0.008 (3)	0.015 (4)	0.002 (3)
C2	0.062 (5)	0.011 (4)	0.045 (5)	0.005 (4)	0.033 (4)	0.009 (3)
C3	0.061 (5)	0.006 (3)	0.050 (5)	−0.002 (3)	0.042 (4)	−0.002 (3)
C4	0.065 (6)	0.021 (4)	0.025 (4)	−0.001 (4)	0.028 (4)	0.000 (3)
C5	0.055 (5)	0.024 (4)	0.030 (4)	0.004 (3)	0.030 (4)	0.001 (3)
C6	0.043 (5)	0.010 (3)	0.038 (4)	−0.002 (3)	0.026 (4)	−0.003 (3)
C7	0.048 (5)	0.011 (4)	0.044 (4)	−0.002 (3)	0.034 (4)	−0.004 (3)
C8	0.045 (5)	0.010 (3)	0.037 (4)	−0.001 (3)	0.030 (4)	−0.001 (3)
C9	0.042 (5)	0.045 (5)	0.039 (4)	0.004 (4)	0.013 (4)	−0.004 (4)
C10	0.041 (5)	0.050 (5)	0.039 (4)	−0.010 (4)	0.019 (4)	−0.009 (4)
C11	0.034 (4)	0.026 (4)	0.051 (5)	0.007 (3)	0.016 (4)	0.001 (4)

### Geometric parameters (Å, °)

**Table:**

Re1—O1	1.698 (4)	C2—H2	0.9500
Re1—O2	1.861 (5)	C3—H3	0.9500
Re1—N1	2.175 (5)	C4—C5	1.395 (10)
Re1—N2	2.183 (6)	C4—H4	0.9500
Re1—I1	2.7176 (5)	C5—C6	1.368 (10)
Re1—I2	2.7235 (5)	C5—H5	0.9500
O2—C9	1.411 (10)	C6—H6	0.9500
N1—C1	1.345 (8)	C7—C8	1.502 (10)
N1—C7	1.353 (9)	C9—C10	1.478 (11)
N2—C6	1.339 (8)	C9—C11	1.526 (10)
N2—C8	1.348 (8)	C9—H9	1.0000
N3—C7	1.326 (8)	C10—H10A	0.9800
N3—C3	1.341 (9)	C10—H10B	0.9800
N4—C8	1.320 (9)	C10—H10C	0.9800
N4—C4	1.337 (9)	C11—H11A	0.9800
C1—C2	1.361 (10)	C11—H11B	0.9800
C1—H1	0.9500	C11—H11C	0.9800
C2—C3	1.380 (10)		
			
O1—Re1—O2	169.68 (19)	N4—C4—C5	122.8 (6)
O1—Re1—N1	88.66 (19)	N4—C4—H4	118.6
O2—Re1—N1	83.34 (19)	C5—C4—H4	118.6
O1—Re1—N2	86.9 (2)	C6—C5—C4	116.6 (6)
O2—Re1—N2	84.9 (2)	C6—C5—H5	121.7
N1—Re1—N2	76.5 (2)	C4—C5—H5	121.7
O1—Re1—I1	94.56 (13)	N2—C6—C5	121.3 (6)
O2—Re1—I1	92.91 (15)	N2—C6—H6	119.4
N1—Re1—I1	97.18 (14)	C5—C6—H6	119.4
N2—Re1—I1	173.47 (14)	N3—C7—N1	125.1 (6)
O1—Re1—I2	97.35 (14)	N3—C7—C8	118.3 (6)
O2—Re1—I2	89.91 (13)	N1—C7—C8	116.6 (6)
N1—Re1—I2	171.11 (14)	N4—C8—N2	125.3 (6)
N2—Re1—I2	97.22 (14)	N4—C8—C7	118.7 (6)
I1—Re1—I2	88.908 (16)	N2—C8—C7	115.9 (6)
C9—O2—Re1	160.7 (5)	O2—C9—C10	110.3 (6)
C1—N1—C7	118.0 (5)	O2—C9—C11	108.4 (7)
C1—N1—Re1	126.7 (4)	C10—C9—C11	114.6 (7)
C7—N1—Re1	115.2 (4)	O2—C9—H9	107.7
C6—N2—C8	117.7 (6)	C10—C9—H9	107.7
C6—N2—Re1	126.8 (4)	C11—C9—H9	107.7
C8—N2—Re1	115.3 (4)	C9—C10—H10A	109.5
C7—N3—C3	115.6 (6)	C9—C10—H10B	109.5
C8—N4—C4	116.2 (6)	H10A—C10—H10B	109.5
N1—C1—C2	120.3 (7)	C9—C10—H10C	109.5
N1—C1—H1	119.8	H10A—C10—H10C	109.5
C2—C1—H1	119.8	H10B—C10—H10C	109.5
C1—C2—C3	117.9 (7)	C9—C11—H11A	109.5
C1—C2—H2	121.1	C9—C11—H11B	109.5
C3—C2—H2	121.1	H11A—C11—H11B	109.5
N3—C3—C2	123.1 (6)	C9—C11—H11C	109.5
N3—C3—H3	118.5	H11A—C11—H11C	109.5
C2—C3—H3	118.5	H11B—C11—H11C	109.5
			
O1—Re1—O2—C9	−37 (2)	C1—C2—C3—N3	−3.1 (11)
N1—Re1—O2—C9	−76.5 (14)	C8—N4—C4—C5	2.4 (11)
N2—Re1—O2—C9	0.5 (14)	N4—C4—C5—C6	0.8 (11)
I1—Re1—O2—C9	−173.4 (14)	C8—N2—C6—C5	2.2 (10)
I2—Re1—O2—C9	97.7 (14)	Re1—N2—C6—C5	176.0 (5)
O1—Re1—N1—C1	100.5 (6)	C4—C5—C6—N2	−3.2 (10)
O2—Re1—N1—C1	−86.0 (6)	C3—N3—C7—N1	2.4 (10)
N2—Re1—N1—C1	−172.3 (6)	C3—N3—C7—C8	−176.6 (6)
I1—Re1—N1—C1	6.1 (6)	C1—N1—C7—N3	−3.6 (10)
O1—Re1—N1—C7	−83.2 (5)	Re1—N1—C7—N3	179.7 (5)
O2—Re1—N1—C7	90.2 (5)	C1—N1—C7—C8	175.4 (6)
N2—Re1—N1—C7	3.9 (4)	Re1—N1—C7—C8	−1.3 (7)
I1—Re1—N1—C7	−177.7 (4)	C4—N4—C8—N2	−3.7 (11)
O1—Re1—N2—C6	−91.0 (6)	C4—N4—C8—C7	175.4 (6)
O2—Re1—N2—C6	95.2 (6)	C6—N2—C8—N4	1.5 (11)
N1—Re1—N2—C6	179.6 (6)	Re1—N2—C8—N4	−173.1 (6)
I2—Re1—N2—C6	6.0 (6)	C6—N2—C8—C7	−177.6 (6)
O1—Re1—N2—C8	83.0 (5)	Re1—N2—C8—C7	7.8 (8)
O2—Re1—N2—C8	−90.8 (5)	N3—C7—C8—N4	−4.5 (10)
N1—Re1—N2—C8	−6.4 (5)	N1—C7—C8—N4	176.4 (6)
I2—Re1—N2—C8	180.0 (5)	N3—C7—C8—N2	174.7 (6)
C7—N1—C1—C2	1.3 (11)	N1—C7—C8—N2	−4.4 (9)
Re1—N1—C1—C2	177.5 (5)	Re1—O2—C9—C10	−130.8 (12)
N1—C1—C2—C3	1.8 (11)	Re1—O2—C9—C11	102.9 (14)
C7—N3—C3—C2	1.1 (10)		

### Hydrogen-bond geometry (Å, °)

**Table:**

*D*—H···*A*	*D*—H	H···*A*	*D*···*A*	*D*—H···*A*
C1—H1···I1	0.95	3.04	3.697 (7)	127
C6—H6···I2	0.95	3.05	3.709 (7)	128
C5—H5···N4^i^	0.95	2.58	3.504 (9)	163
C6—H6···O1^ii^	0.95	2.53	3.193 (9)	127
C9—H9···I1^iii^	1.00	3.02	3.834 (8)	139

Symmetry codes: (i) −*x*+1, *y*−1/2, −*z*+1/2; (ii) −*x*+1, −*y*−1, −*z*; (iii) *x*, −*y*−1/2, *z*+1/2.
